# Towards robot-assisted magnetic particle imaging for mobile point-of-care (neuro)vascular applications

**DOI:** 10.1038/s41598-026-61392-y

**Published:** 2026-07-06

**Authors:** Matthias N. van Oosterom, Aran van Welzen, Leon J. Slof, Adriaan C. G. M. van Es, Andrew G. Webb, Stewart Bartlett, Aidan Cousins, Nyika D. Kruyt, Jos H. G. M. Haverkamp, Fijs W. B. van Leeuwen

**Affiliations:** 1 Interventional Molecular Imaging Laboratory, Leiden University Medical Center, Leiden, The Netherlands; 2 Design and Prototyping, Department of Medical Technology, Leiden University Medical Centre, Leiden, The Netherlands; 3 Department of Radiology, Leiden University Medical Center, Leiden, The Netherlands; 4 Department of Radiology, C.J. Gorter MRI Center, Leiden University Medical Center, Leiden, The Netherlands; 5 Ferronova Pty Ltd, Adelaide, Australia; 6 Department of Neurology, Leiden University Medical Center, Leiden, The Netherlands

**Keywords:** Emergency services, Paramedic, Robotic imaging, Stroke, Neurovascular imaging, Magnetics, Ambulance, Engineering, Neuroscience

## Abstract

Rapid diagnosis, timely treatment, and continuous monitoring are essential for improving outcomes in neurovascular diseases (time=brain). Such point-of-care applications, however, require imaging to be conducted in dynamic and often confined spaces. While magnetic particle imaging (MPI) shows preclinical promise for fast (neuro)vascular imaging, it relies on complex and restricted imaging gantries. Here, we present a unique gantry-free robot-assisted MPI strategy (Robotic MPI) that is ‘portable’. Using permanent magnets, Hall sensors and correction coils, we developed a single-sided detector with a detection threshold of ~ 5 µg iron-containing contrast agent at the detector surface. Robot-assisted positioning and pose tracking, combined with a dedicated maximum likelihood expectation-maximization-based reconstruction algorithm, translates detector signals into tomographic images with a spatial resolvability of 7 mm (visually evaluated at an imaging depth of ~ 5 mm). Surface imaging (SI) provided anatomical reference, delivering hybrid MPI/SI scans. Robotic-MPI’s ability to visualize 3D (neuro)vasculature in a phantom model, highlights the system’s potential for next-generation, portable vascular imaging solutions.

## Introduction

Neurovascular diseases such as stroke—including ischemic and hemorrhagic subtypes—and traumatic brain injury are the primary cause for disability and the second most common cause of death worldwide^1–3^. Of these, stroke is among the most common (1-case every 3-seconds worldwide) and impactful (143-million years of disability-free life lost each year)^1–3^. As stroke results from arterial occlusion or rupture, cerebral blood flow is disrupted and neuronal death is rapidly induced —approximately two million neurons per minute (“time = brain”)^4^. In addition, acute treatments differ between stroke subtypes, rendering correct patient referral and allocation a serious concern; for example, patients eligible for endovascular thrombectomy (EVT) should be transported to an EVT-capable center, whereas infarctions without proximal vessel occlusion can be managed at the nearest stroke center, and (in future) patients with hemorrhagic stroke should be brought to a center with neurosurgical capability. Consequently, rapid diagnosis, timely treatment, and continuous monitoring are considered essential to improve patient outcomes^3,5,6^.

Computed tomography (CT) and magnetic resonance imaging (MRI) remain the clinical standards for neurovascular diagnosis and monitoring^7^. In some urban centers, dedicated ambulance trucks are equipped with CT scanners (called mobile stroke-units) to even enable out-of-hospital stroke imaging^8–10^. Similar concepts are being proposed for mobile MRI as well^11^. Both mobile CT and MRI require large gantry-based systems, typically weighing *≥* 500 kg^11–14^. This severely limits portability and use in critical care settings such as paramedic services, emergency departments, or resource-limited healthcare environments. Moreover, despite being quick (~ 1–2 min for non-contrast CT and up to ~ 8 min for contrast-enhanced protocols), CT exposes patients (and staff) to ionizing radiation, restricting repeated or long-term monitoring, while MRI is costly, time-intensive (typically ~ 10–20 min) and often inaccessible in the acute phase of care. Portable ultrasound and optical-spectroscopy approaches have been proposed as alternatives for neurovascular imaging, but their diagnostic utility is severely limited by skull-induced acoustic and optical attenuation^15,16^. As a result, there remains a clear need for “portable”, radiation-free imaging technologies that can detect neurovascular disruptions through the skull in the point-of-care setting.

Magnetic Particle Imaging (MPI) directly detects the magnetization response of superparamagnetic iron oxide nanoparticles (SPIONs), providing high temporal resolution and quantitative imaging without background tissue signal^17,18^. Traditionally, this is achieved by generating strong magnetic field gradients to dynamically move a field-free region through the object within which the SPION contrast agent is detected. Since its introduction by Gleich and Weizenecker in 2005^19^, MPI has demonstrated promise across diverse preclinical applications, including cerebral perfusion imaging, cardiovascular interventions, and inflammation tracking^20–22^. Unlike CT or MRI, MPI enables dynamic, repeated imaging without radiation or endogenous background signal, making it an attractive candidate for point-of-care neuroimaging. Unfortunately, full-body human MPI systems are currently not commercially available due to challenges in scaling the imaging gantry, particularly with respect to maintaining sufficiently strong magnetic field gradients and receiver sensitivity while managing heat dissipation and power constraints^18,23,24^. Consequently, translational advances have focused on smaller region-specific systems^25^; a ~ 2 × 2 m head scanning system proposed by Mattingly et al.^26^, a ~ 1 × 0.5 m head scanning system introduced by Graeser et al.^27^ and Thieben et al.^28^, and a ~ 0.5 × 0.5 m lower-limb perfusion scanner by Vogel et al.^29^ and Hartung et al.^30^. While these gantry-based prototypes represent promising technological advances, their fixed geometries inherently restrict imaging to specific anatomical regions, limiting flexibility for broader deployment. As such, several groups have worked towards single-sided open MPI configurations, including early demonstrations of 3D tomographic imaging at limited scale^31–33^. As these approaches rely on conventional field-based spatial encoding, the effective field-of-view remains constrained by the system geometry, requiring increasingly large configurations for larger imaging volumes. Although only demonstrated on a small scale yet (~ 2.5 × 5 cm), Wang et al. further demonstrated that motorized grid-scanning with such a single-sided system, similar as in a 3D printer, could theoretically extend the scanned volume, dependent on the size of the motorized stage^34^. These developments highlight the continued need and scientific interest to explore MPI strategies that are compact and portable, while being region-agnostic, enabling 3D tomographic imaging outside dedicated gantry and single-sided setups, for example in out-of-hospital emergency settings such as ambulances or trauma helicopters.

Image efforts in dynamic and space restricted surgical environments have indicated that ‘gantry-free imaging’ using portable 0D-, 1D-, or 2D-detectors can provide diagnostically valuable 3D tomographic images not restricted to a specific field-of-view^35,36^. In such systems, tracking technologies record the detector’s position and orientation (pose) during data acquisition, and dedicated reconstruction algorithms combine this information with detector response models on-the-fly, allowing for fully flexible and adaptive acquisition geometries. This concept has been successfully explored for various tracer signals, including gamma^37–39^, beta^40^ and fluorescence^39^. Critically, we have previously shown that even 0D magnetic particle detection probes, in combination with an optical tracking system, can be used to image SPION accumulation in sentinel lymph nodes of porcine surgical models^41^. This opened a novel approach for gantry-free MPI solutions employing spatial sampling as the primary driver for 3D tomographic reconstruction, in contrast to conventional field-based spatial encoding. A limitation for the dissemination of this concept to a point-of-care setting is the optical tracking (i.e., direct line-of-sight needs to be maintained). For such applications mechanical tracking would be more reliable, as well as support detector scalability and further automation, offering a promising alternative to conventional gantry-based imaging.

**Fig. 1:**
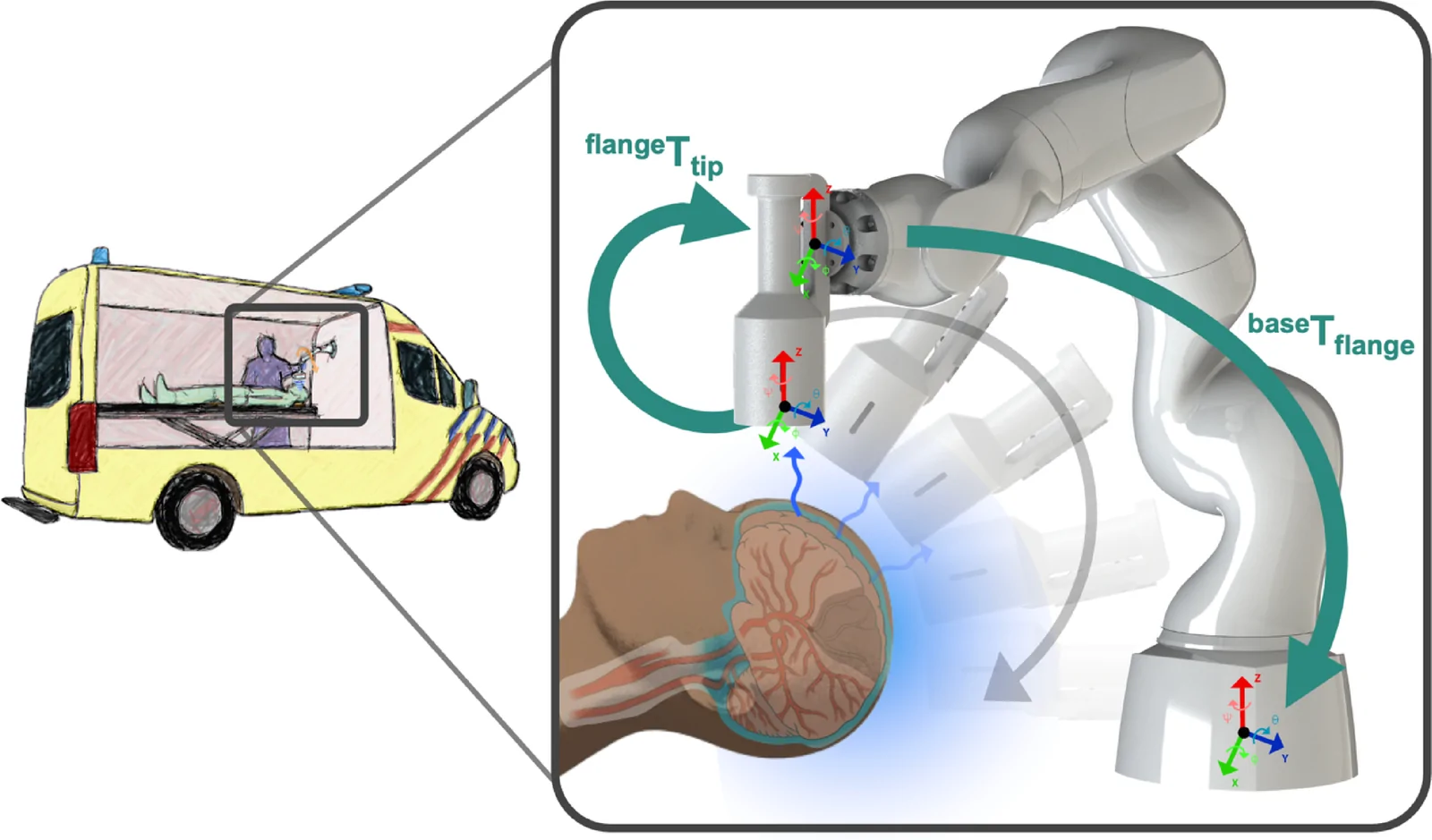
Robot-assisted MPI concept in the ambulance setting. The MPI system consists of a single-sided SPION contrast detector mounted on a robotic arm. Using the underlying pose transformations (in green), the robot tracks the detector tip’s position and orientation as it rotates around the imaging object. Combining detector readout, modeling and pose tracking then forms the basis for dedicated reconstruction algorithms, generating the 3D scan of the contrast agent. (Illustrations created by the authors using SOLIDWORKS 2022 [https://www.solidworks.com] and Adobe Illustrator/Photoshop 2025 [https://www.adobe.com]).

Building on these advances, we present a novel robot-assisted MPI system (‘Robotic MPI’) that has a small form factor and the ‘portability’ needed to be installed in e.g. ambulances (Fig. 1). The developed prototype relies on a single-sided field scanner that is mechanically tracked, thus enabling flexible imaging geometries that can adapt to the target size at hand. Where conventional MPI relies on detecting nonlinear SPION magnetization within the dynamically manipulated field-free region, the presented system investigates a fundamentally different acquisition and reconstruction framework based on robotic spatial sampling, static SPION magnetization, magnetic field sensing and filtering, detector modeling and dedicated iterative reconstruction for 3D tomographic imaging. Here, we detail the system architecture, magnetic field design, calibration method, reconstruction algorithm and initial phantom performance. Thereby indicating the unique potential of Robotic MPI, especially in the context of point-of-care neurovascular imaging.

## Results

### System overview

The Robotic MPI system (Fig. 1) consists of four main components: (1) a custom single-sided 0D SPION contrast detector with highly amplified signal read-out; (2) a robotic arm (LBR-med 14, KUKA GmbH) with embedded mechanical position and orientation tracking; (3) on-the-fly tomographic image reconstruction via a dedicated maximum likelihood expectation-maximization (MLEM) based algorithm; (4) scan fusion with optical surface imaging for anatomical reference. These individual components are described in the sections below, while further product and phantom detailing can be found in the Methods section.

**Fig. 2:**
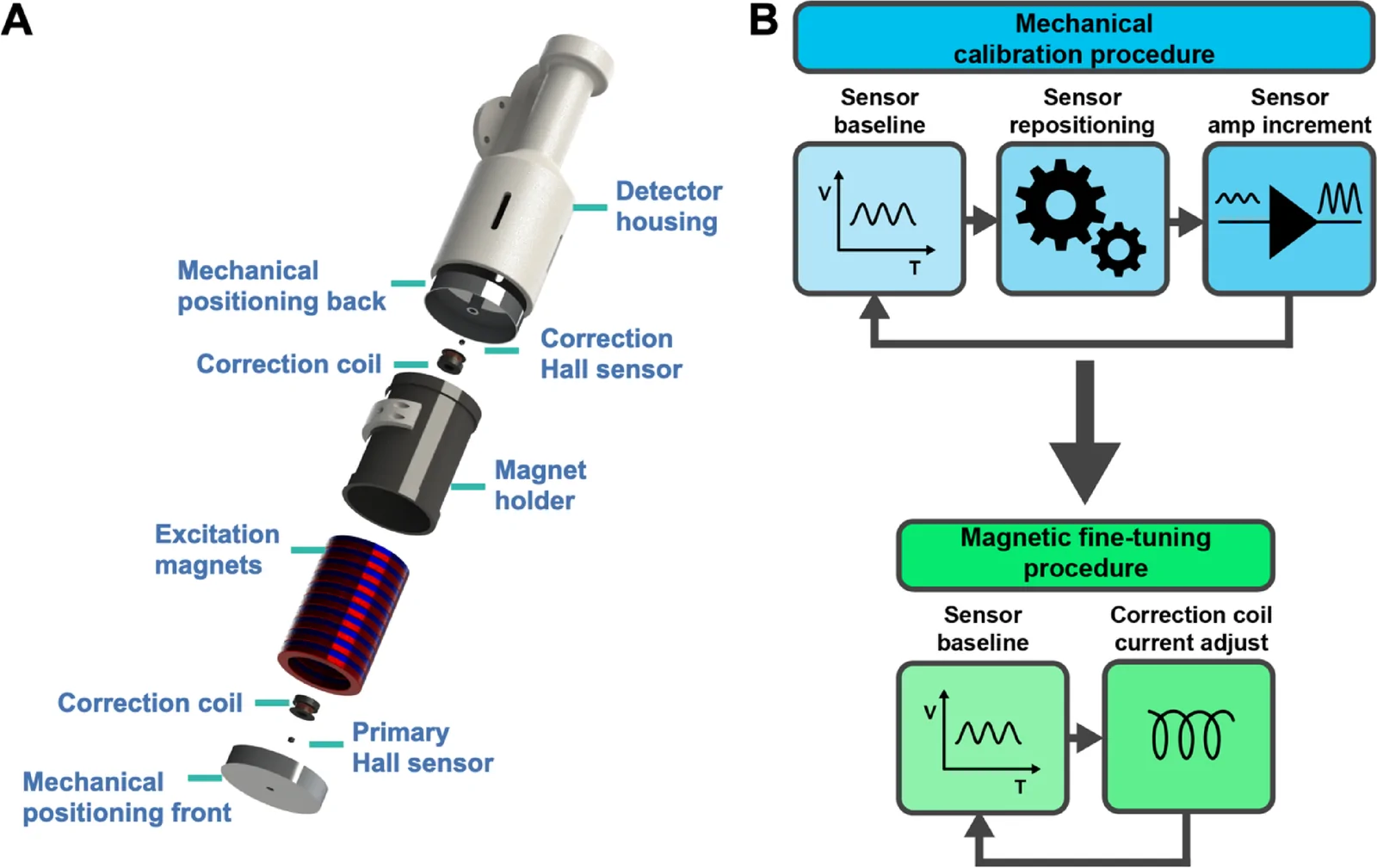
Detector design and calibration procedure. (**A**) Schematic illustration of the hardware components housed within the single-sided detector. (**B**) Schematic of the iterative mechanical calibration procedure, followed by the magnetic fine-tuning procedure, enabling significant sensor read-out amplification while minimizing any contribution of the excitation magnets to the signal. (Illustrations created by the authors using SOLIDWORKS 2022 [https://www.solidworks.com] and Adobe Illustrator 2025 [https://www.adobe.com]).

#### Detector, calibration and amplification

**Fig. 3:**
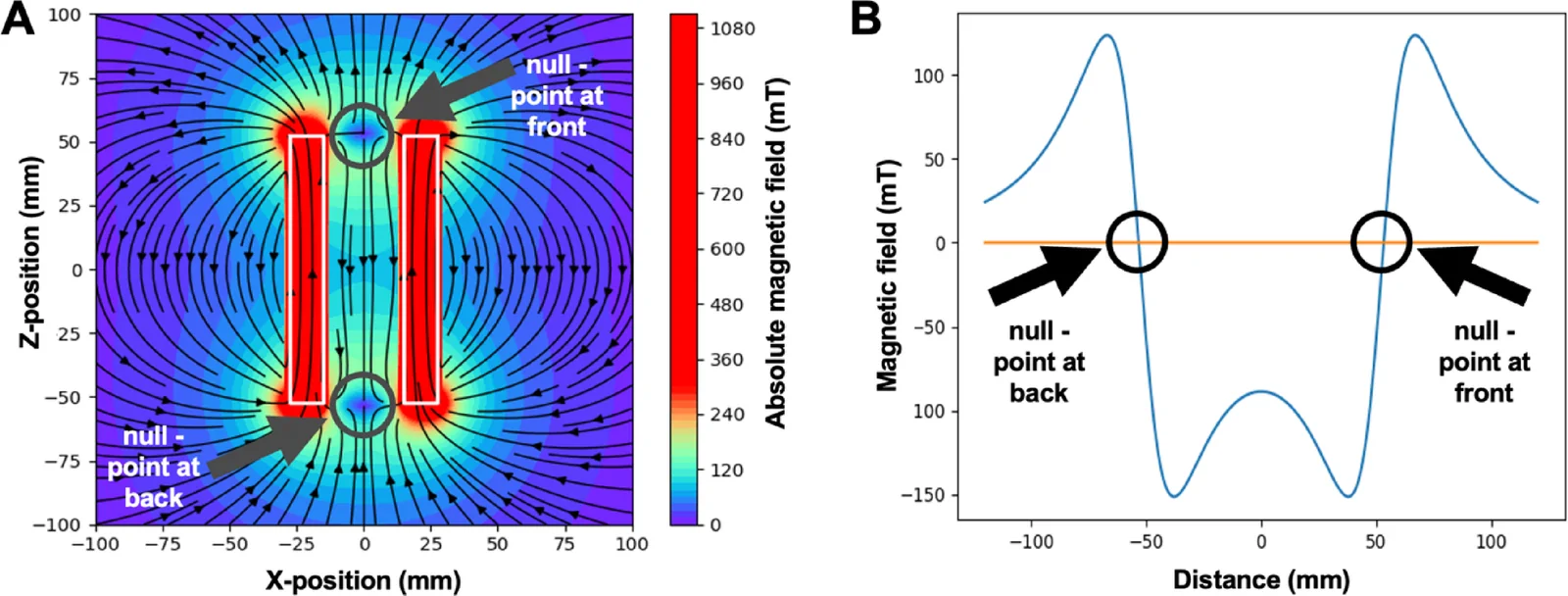
Characterization of the detector magnetic excitation field. (**A**) Simulation of the magnetic excitation field (Magpylib library) showing a x-z slice through the middle of the magnet cylinder housed within the detector. The gray arrows and white text indicate the null-magnetic field points at the front and back of the magnet. (**B**) A plot of the magnetic field through the longitudinal axis of the magnetic excitation field, indicating the steep slope around the null-magnetic field points at the front and back of the magnet setup. The detector Hall sensors are positioned in these null-magnetic field points.

To prevent the need for high power (i.e., high current flows), while using relatively low cost and readily available materials, the detector (Fig. 2) is based on a permanent magnet approach^42^. This approach leverages the unique magnetic field-line properties of ring-shaped magnets that exhibit small, but definite, null-magnetic field points at both the front and back of the ring (Fig. 3). Precise placement of a Hall sensor in a null-magnetic field point made the field measured from the excitation ring magnets approach zero. This allowed for the Hall sensor to measure the magnetic fields created by SPION contrast agents upon excitation with the ring magnets. The signal intensity was influenced by the amount of SPIONs and the distance to the sensor. By placing a Hall sensor in both the front and back null-magnetic field points (Fig. 3B), ambient magnetic fields (i.e., the earth’s magnetic field) could be compensated for by subtracting sensor 2 (background only) from sensor 1 (contrast agent + background).

**Fig. 4:**
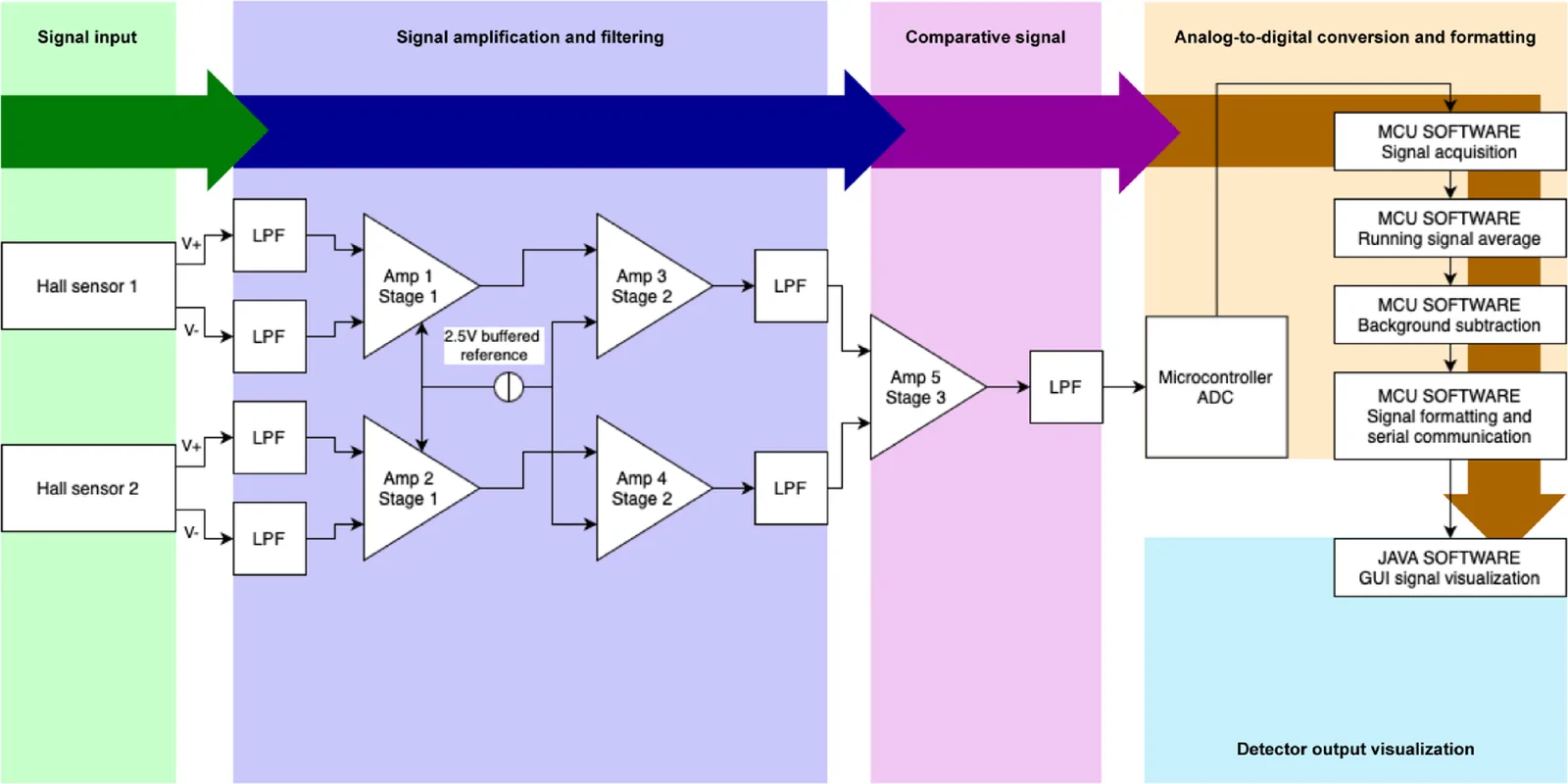
Overview of　the numerical detector read-out. The read-out contains dual-sensor input (green), 3 stages of amplification and low-pass filtering (purple and pink), sensor signal comparison (pink), analog-to-digital conversion (orange), digital processing (orange) and count based visualization (blue). Overall signal flow is denoted with the large block arrows. Amp, amplifier; LPF, low-pass filter; ADC, analog-to-digital converter; MCU, microcontroller and GUI, graphical user interface.

 Detector geometry and precise sensor positioning turned out to be critical for sensitive separation of contrast-induced and excitation-induced magnetic field readings, and as such, the detector sensitivity. First of all, the choice of magnet geometry had substantial influence. To decreases the slope of the magnetic field around the null-magnetic field points the ratio between the outer diameter and the inner magnet diameter was optimized using computational simulations (Magpylib Python libraries^43^ and commercial availability (Fig. 3). This resulted in the following magnet setup: 15 Neodymium rings with a magnetization of 1.17 T stacked (all orientated in the same direction). Each ring has an outer diameter of 55 mm, inner diameter of 30 mm (ratio 1.8:1) and a thickness of 7 mm. The total 105 mm long arrangement provides a slope of 17.8 mT/mm around the individual null-magnetic field points. This arrangement is enclosed in a plastic (PVC, CNC milled) housing. Secondly, fine mechanical sensor calibration had to be enabled. The 2 Hall sensors were individually fixed in aluminum caps (CNC milled) that can be screwed on the plastic magnet housing (Fig. 2A). A fine thread with a 0.5 mm pitch allowed for a ‘micro-meter’ precise sensor displacement (i.e., 1.4 μm/^o^) along the longitudinal axis of the magnet arrangement, allowing for an iterative mechanical calibration method (Fig. 2B). Here, the sensor is mechanically placed in the null-magnetic field point based on the direct sensor read-out signal, starting out with a low level of signal amplification. After such a mechanical positioning step, the sensor signal is further amplified, revealing any remaining position misalignment, which can then again be corrected for mechanically. Following several positioning and amplification steps, this allowed for a total signal amplification of 92 dB, using a 3-stage amplifier setup (Fig. 4, see detailing in the Methods section). As a third optimization, a correction/compensation coil was placed at the individual Hall sensors, allowing for any last fine tuning of the magnetic field after complete assembly of the detector arrangement (12 turns consisting of 2 layers of 6; see Fig. 2). Using a small current (order of 50–200 µA), these coils helped to compensate for any measurable deviation of the sensors with their respective null-magnetic field points.

The entire detector setup was mounted in a plastic (PLA, FDM 3D printed) housing (diameter 95 mm, height 272.5 mm), that includes a handle on top to easily move the detector, and a mounting plate for connection to the robotic arm (described below).

#### Robot-assisted acquisition

The detector was mounted on a robotic arm (Fig. 1), which could be moved either by hand (i.e., robot-assisted, using a foot pedal to engage hand guiding), or automatically. The arm enabled convenient movement of the detector around the subject of interest, while tracking its pose (6 degrees of freedom: x, y, z, roll, pitch, yaw) for every individual measurement during the acquisition. As such, acquisition consisted of moving the detector to cover the suspected volume of interest with as many unique poses as possible within a chosen acquisition time. To allow pose calculation, the detector tip was calibrated (Fig. 1) with respect to the flange (i.e., the end effector) of the robot using the known geometry as measured in the CAD models of the housing. This resulted in pose monitoring of the detector tip within the coordinate system of the robot base (i.e., ^base^T_tip_ = ^base^T_flange_ x ^flange^T_tip_; Fig. 1), providing a dataset of detector measurements with synchronized pose. Before using this dataset for reconstruction, the data was preprocessed by averaging near-duplicate measurements, while interpolating acquisition steps larger than 1 mm (i.e. linear interpolation) and filtering (i.e., Hampel filter with 5 mm window and sigma, a 3 mm median filter and 0.3 mm Gaussian filter).

#### Iterative scan reconstruction

Dedicated tomographic reconstruction algorithms helped solve the reconstruction challenges associated with freehand imaging methods (i.e., imaging without a fixed acquisition geometry)^39,44^. As the presented robotic MPI approach operates with a flexible scan geometry and therefore without a fixed system matrix, a scan-specific system matrix (also referred to as the system function) is constructed based on a measured detector response model (i.e., look-up table), transformed according to the recorded pose of each individual detector measurement. The look-up table was generated with robotic wet-lab measurements, described in the Methods section at the end of the article. The resulting reconstruction algorithm subsequently combines the individual detector measurements, detector poses and the pose-matched look-up tables within an iterative maximum-likelihood expectation-maximization (MLEM) framework.

Among the tested MLEM approaches, the following algorithm yielded the best results in capturing high-intensity regions while suppressing noise and artifacts in low-intensity areas and maintaining resolvability (as characterized in the following section). The algorithm builds the volumetric field $\:V\left(\mathbf{r}\right)$ from measured signal counts $\:{c}_{i}$ by alternating between forward projection, multiplicative update and a total variation (TV) regularization step using 15 iterations. The number of iterations was chosen empirically, as reconstructions showed convergence in terms of structural appearance and intensity distribution within this range, while additional iterations resulted in negligible changes but increased computation time.

Starting with a reconstruction prior restricted to locations within reach of the detector, each iteration calculates the estimated detector signal for each measurement * i* as:

1 $$\hat{c}_{i}^{{\left( k \right)}} = \sum\limits_{{\mathbf{r}}} {V^{{\left( k \right)}} \left( {\mathbf{r}} \right)\,w_{i} \left( {\mathbf{r}} \right)}$$

where $\:{w}_{i}\left(\mathbf{r}\right)$ represents the pose-matched look-up table defining the contribution of voxel $\:\mathbf{r}$ to measurement * i*. $\:{V}^{\left(k\right)}\left(\mathbf{r}\right)$ denotes the reconstructed voxel intensity at iteration * k* and $\:{\widehat{c}}_{i}^{\left(k\right)}$ the estimated detector counts. The reconstructed volume is then updated multiplicatively according to the ratio between measured and estimated counts:

2 $${V^{{\left( {k + 1} \right)}} \left( {\mathbf{r}} \right) = V^{{\left( k \right)}} \left( {\mathbf{r}} \right) \cdot \frac{{\mathop \sum \nolimits_{i} w_{i} \left( {\mathbf{r}} \right)\,\frac{{c_{i} }}{{\hat{c}_{i}^{{\left( k \right)}} }}}}{{\mathop \sum \nolimits_{i} w_{i} \left( {\mathbf{r}} \right)}}}$$

where $\:{c}_{i}$ represents the measured detector counts at measurement * i*. To strengthen true-negative readings, low count measurements are treated with a minimal negative contribution to avoid biasing of the reconstruction. After each update, a mild isotropic total variation (TV; Chambolle algorithm) regularization is applied to suppress small-scale noise while preserving sharp structural boundaries:

3 $$\tilde{V}^{{\left( {k + 1} \right)}} \left( {r} \right) = \arg \mathop {\min }\limits_{U} \;\sum\limits_{{r}} {\left[ {\frac{1}{2}\left( {V^{{\left( {k + 1} \right)}} \left( {r} \right) - U\left( {r} \right)} \right)^{2} + \lambda \sqrt {\left( {\nabla _{x} U\left( {r} \right)} \right)^{2} + \left( {\nabla _{y} U\left( {r} \right)} \right)^{2} + \left( {\nabla _{z} U\left( {r} \right)} \right)^{2} } } \right]}$$

Where $\:{\stackrel{\sim}{V}}^{(k+1)}\left(\boldsymbol{r}\right)$ denotes the TV regularized version of the reconstruction volume $\:{V}^{(k+1)}\left(\mathbf{r}\right)$ obtained after MLEM iteration * k+1*,$\:U\left(\boldsymbol{r}\right)$ denotes a candidate image reconstruction evaluated during the TV minimization process and $\:\lambda\:$ controls the strength of the TV regularization (higher values providing stronger denoising). Here, the first term enforces agreement with the MLEM reconstruction, whereas the second term penalizes large spatial gradients to suppress noise while preserving object boundaries. The regularization weight (*λ* =1.25) was determined empirically through qualitative evaluation, balancing noise reduction against preservation of anatomical and structural image detail.

#### Hybrid MPI solution using 3D surface imaging—robotic MPI/SI

**Fig. 5:**
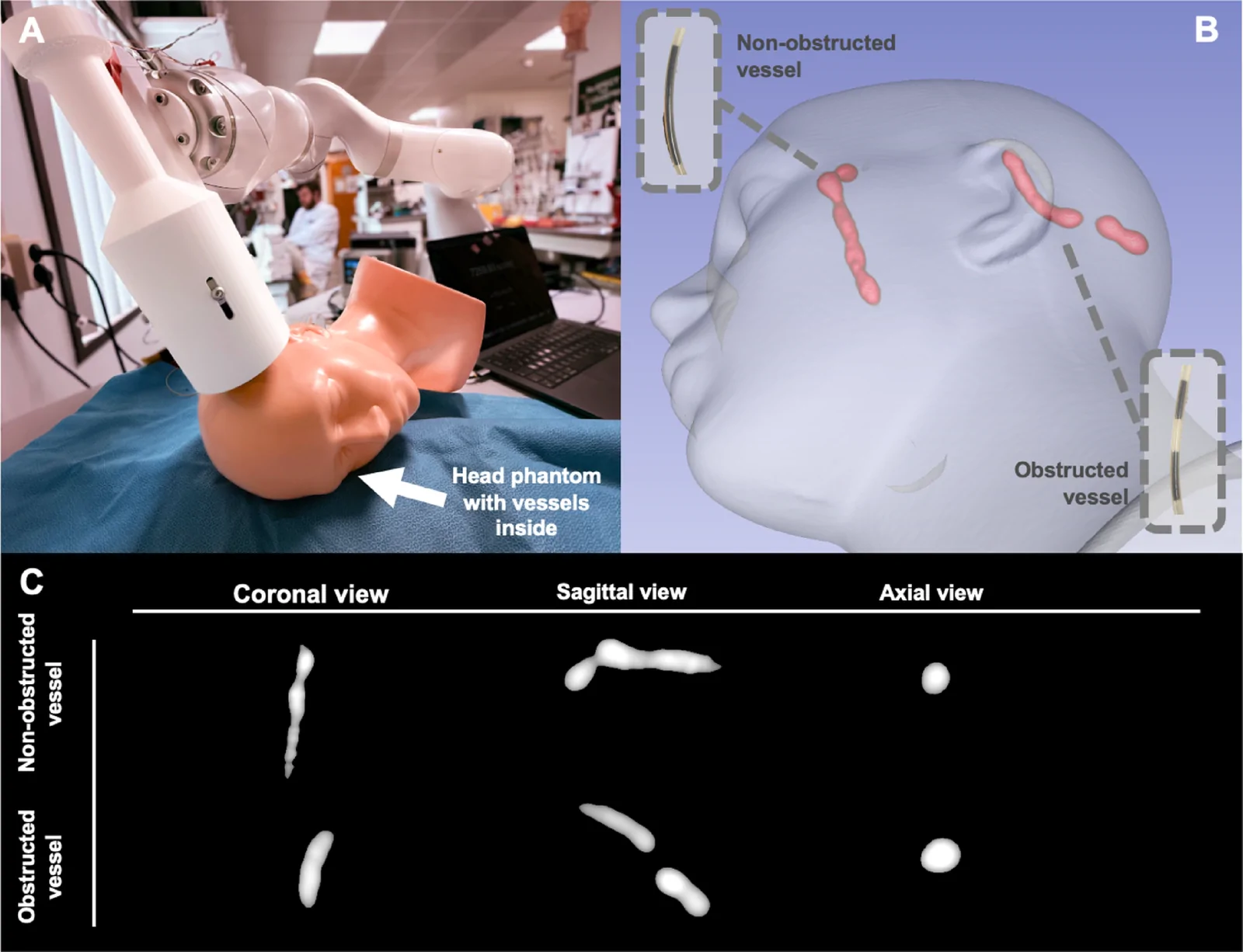
Proof-of-concept – gantry free robotic MPI/SI for point-of-care neurovascular imaging. (**A**) Initial phantom validation setup, presenting the robotic MPI system and a hollow head phantom with two vessels inside. (**B**) 3D volume rendering of the resulting MPI/SI reconstruction, able to detect both the non-obstructed and the obstructed vessels, visualized with respect to the phantom skin. (**C**) Coronal, sagittal and axial slices through the MPI tomographic reconstruction, displaying different slices for the non-obstructed and the obstructed vessel. (Visualizations created by the authors using 3D Slicer 5.8.1 [https://www.slicer.org] and the Napari Python image viewer [https://napari.org].).

The resulting MPI reconstruction was successfully fused with a 3D model of the subject of interest using optical surface imaging (detailed in the Methods section). This enabled evaluation of the MPI images within the reference frame of the phantom skin (Fig. 5), improving anatomical appreciation by providing a hybrid modality in the form of Robotic Magnetic Particle Imaging/Surface Imaging (Robotic MPI/SI).

### System proof-of-concept

Figure 5 demonstrates results of the complete Robotic MPI/SI system, showing successful acquisition and 3D tomographic reconstruction in a neurovascular phantom setting. The complete system weight is ~ 50 kg, of which the robot arm is ~ 30 kg, the robot computer is ~ 20 kg and the detector is only ~ 2 kg. The acquisition time and volume were adapted to the targets at hand. During acquisition of this specific phantom, the detector was moved to as many unique positions and orientations around the object surface as possible within a ~ 1.5 min acquisition time. The reconstruction time, running on a MacBook M3 pro CPU (Apple inc.) without GPU acceleration, took ~ 1 min. Figure 5 demonstrates the ability to distinguish between vessel shapes within the head phantom and showing them in context of the skin surface. One vessel (length 90 mm; diameter 3.2 mm; 19 mg SPION) represented a ‘normal’ vessel (‘non-obstructed vessel’), the other vessel (length 90 mm; diameter 3.2 mm; 14 mg SPION) held a clear cold spot (25 mm length) representing interrupted flow (‘obstructed vessel’).

### System sensitivity and resolvability

**Fig. 6:**
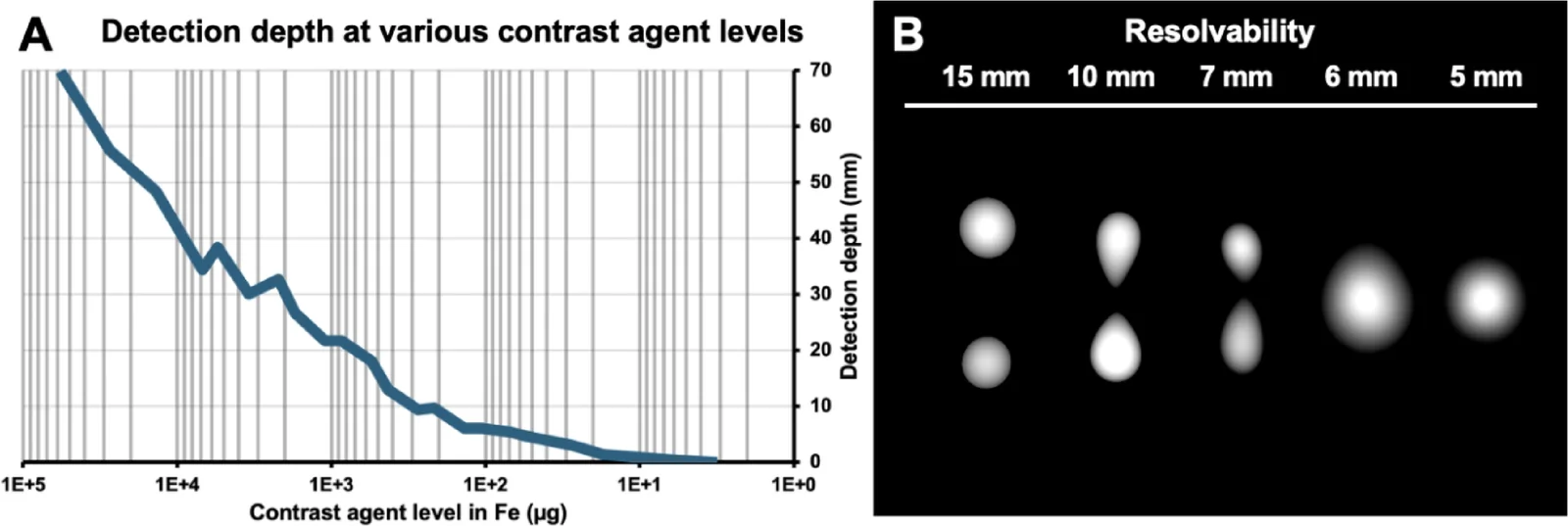
MPI depth penetration, sensitivity and resolvability. (**A**) Overview of the detector detection depth as a function of contrast agent levels, expressed in the amount of detectable iron mass; as such also providing the sensitivity limit at every imaging depth. (**B**) Overview of the resolvability limits of the current MPI system demonstrated using two 0.8 mm inside diameter capillaries filled with FerroTrace.

Figure 6A visualizes both the detection depth and sensitivity of the Robotic MPI system (see Methods for setup detailing). These showed to be directly related to each other, where increased source depth resulted in decreased sensitivity. With the current detector prototype, depth detection reached beyond 70 mm if > 55 mg iron contrast agent was present. This detection depth dropped with lower iron contents, indicating a current limit of detection at ~ 5 µg iron contrast agent right at the detector surface. Figure 6B shows the resolvability of the Robotic MPI prototype at shallow imaging depth (within 5 mm; see Methods), showing that sources as close as 7 mm (edge-to-edge) could still be visually distinguished from each other in the current reconstruction.

## Discussion

Within this study we present a Robotic MPI prototype that can detect vascular occlusions in a head phantom, with an edge-to-edge system resolvability of 7 mm (visually assessed at a ~ 5 mm imaging depth). The gantry-free design enables a relatively light and portable system, not limited to a specific acquisition trajectory or anatomy. Uniquely the physiological information can be placed in anatomical context using 3D surface scanning, effectively yielding the first hybrid MPI/SI imaging modality. Combined, this puts forward engineering innovations that could help translate MPI to a point-of-care (neuro)vascular imaging modality for dynamic settings, e.g., in ambulances.

Successful translation of MPI into clinical neurovascular imaging, as explored in this work, will require comprehensive validation across (stages of) disease phenotypes, including sensitivity, specificity, diagnostic thresholds, and required SPION contrast levels. Particular attention should be given to distinguish acute ischemic from hemorrhagic events and to compare MPI with established modalities such as CT and MRI. Nonetheless, given the demonstrated potential, MPI remains a highly promising candidate for non-ionizing and noninvasive (neuro)vascular imaging, justifying successive research toward clinical translation.

While the phantom experiments demonstrate the feasibility of detecting neurovascular structures under controlled conditions, several challenges remain for in-vivo translation, inviting continued systematic research. The proposed reconstruction framework was not quantitatively tested for potential sources of uncertainty. These include tracking accuracy, detector-tip calibration accuracy, sparse or uneven spatial sampling, interpolation across larger acquisition gaps and deviations between the measured detector response model (i.e., look-up table) and the actual detector response during scanning. Such effects may influence reconstruction quality through reduced resolvability, geometric distortion and reduced contrast-to-noise ratio. Related to this, the present experiments were performed using static phantoms and therefore do not account for patient motion, which may introduce image blurring artifacts. Such effects could potentially be mitigated through head fixation or real-time patient-tracking approaches, as often used in freehand imaging settings^41^. In addition, the current phantom setup does not account for surrounding anatomical structures such as the skull, although tissue and bone attenuation are not expected to substantially affect MPI due to the high penetration of the low-frequency magnetic fields employed^45^. Furthermore, the static and homogeneous tracer distributions used in the phantom experiments do not fully represent in-vivo conditions, where vascular contrast is influenced by blood-flow kinetics. Building on established CTA and CE-MRA literature^46^, future studies could evaluate system performance and clinical validity under realistic tracer flow conditions, especially with respect to system acquisition times. With the currently reported acquisition time of approximately 1.5 min for the phantom head scan, such imaging durations may be clinically justifiable within a point-of-care triage workflow perspective, where early diagnosis can significantly reduce time-to-treatment. For example, small-scale out-of-hospital CT imaging studies have demonstrated reductions in time-to-treatment on the order of 40 min when early diagnosis is available for triage decision making in stroke patients^10^. Finally, the achievable tracer concentration in human neurovascular MPI remains a key determinant of clinical feasibility. Since patient data for MPI are currently limited, expected contrast agent levels in human remain uncertain. However, an interesting source of reference is the intravenous use of ferumoxytol SPIONs for patients with iron deficiency, prescribing a single IV dose of 510 mg Fe, followed by a second dose of 510 mg Fe 3–8 days later^47,48^. Assuming a total blood volume of 5 L and a brain blood volume of 100 mL, such a single dose would result in an approximate transient neurovascular contrast uptake of about 10 mg and a concentration of 100 µg/mL, representing an upper-bound estimate under idealized conditions. In practice, vascular concentrations may also vary with bolus administration, imaging timing and patient-specific hemodynamics. With the current detector, this corresponds to detection depths of up to ~ 45 mm, and, using a similar vessel size as in the head phantom examined, ~ 10 mm.

Compared to other 3D tomographic human-sized MPI systems proposed in the literature, the current system demonstrates sensitivity and resolvability within a comparable range of 1–10 µg Fe and 2–7 mm, respectively^26,28–30,34,41^. However, in single-sided geometries, sensitivity (and consequently resolvability) typically decreases with increasing stand-off distance from the detector^34^. Consequently, while gantry-based human MPI systems maintain sensitivity and spatial resolution throughout substantially larger imaging depths, the proposed gantry-free robotic setup currently provides comparable performance primarily for shallow localized imaging, while specifically enabling anatomy-independent and flexible positioning. Further research into detector component design could help increase sensitivity also at larger imaging depths; here recent development of ~ pT-sensitive magneto-resistive sensors might enable research into higher sensitivity single-sided designs^25,49^, as compared to the current hall sensor-based design (~ hundreds of nT sensitivity). Nonetheless, when placing the current spatial resolvability in the context of the envisaged point-of-care neurovascular application, the ability to identify larger vascular occlusions or perfusion deficits may already provide clinically actionable information for patient triage, such as directing patients to a comprehensive stroke center versus the nearest hospital. In this context, it is worth noting that larger clot burdens (e.g., on the order of ~ 8 mm and above) are more likely to require endovascular thrombectomy in specialized stroke centers^50^.

Despite requiring further research and development, the current prototype indicates exciting developments for MPI scanning, including both technical and clinical directions. First, building on a robotic platform, the current MPI prototype opens the possibility of fully automated scanning in the future, reducing operator burden^51^. Since inadequate spatial and angular coverage can reduce reconstruction quality^52^, automated scanning could also reduce user-dependent acquisition variability, ensuring standardized acquisition and improving reconstruction consistency. In addition, as demonstrated, combined with surface imaging, it provides a readily available method to integrated hybrid imaging for MPI (i.e., morphological and anatomical information), without the need of a full-sized CT or MRI^53,54^. Full integration and calibration of this surface imaging with the robotic platform would in the future allow for a fully automatic MPI/SI image registration and fusion, given the fixed relationship in coordinate systems. Furthermore, demonstrating the possibility of gantry-free imaging for an out-of-hospital setting, also holds great potential for other dynamic environments, such as intensive care units, emergency departments and the operating room^41^.

## Conclusion

This study successfully demonstrates a technology pathway towards Robotic MPI, proving its function on a human-sized scale and exploring the first steps towards an application in point-of-care (neuro)vascular imaging. Further steps towards in vivo translation are needed to demonstrate its clinical potential in diseases like stroke and traumatic brain injury.

## Materials and methods

### SPION contrast agent FerroTrace

The SPION contrast agent used in this study is FerroTrace (Ferronova, Adelaide, Australia), a novel SPION contrast agent currently running in various clinical trials for MRI and surgical mapping of sentinel lymph nodes^55^. The vial contains 1 mL with an iron content concentration of 55 mg/mL, consisting of maghemite (-Fe_2_O_3_) iron oxide nanoparticle cores, coated with stabilizing polymers for improved systemic biocompatibility and clearance^56,57^.

### Detector electronics, signal processing and software algorithms

The electronic readout of the detector (Fig. 4) consists of two Hall sensors (HW-102 A, Asahi Kasei Microdevices) connected to a 3-stage amplification with a 3-stage low-pass filtering setup (cut-off at ~ 30 Hz). It is digitized with a microcontroller (Arduino Portenta H7, Arduino, Monza, Italy) and the signal is filtered directly with an exponential running average. Subsequently, background signal is measured by pressing a hardware button connected to the microcontroller when no contrast agent is present. A serial output (set at about 10 Hz) is then conditioned to pass a background corrected signal output, if the measurements are 1.5 times the measured background signal, otherwise passing a value of 0. The serial output is read-in and visualized on a laptop with a simple graphical user interface (GUI) build with JavaFX (Oracle corporation, Austin, Texas, USA). The read out of the numerical detector is shown on a laptop (MacBook Pro M3) during MPI acquisition.

### Reconstruction algorithm and look-up table acquisition

The MLEM reconstruction algorithm is built using Python 3.9.6 (Python Software Foundation, Wilmington, Delaware, United States), using numpy, scipy, matplotlib, plotly, skimage and nibabel libraries. The size of the reconstruction volume was directly dependent on the acquisition geometry chosen during scan acquisition. To avoid edge effects in the scan, this volume was slightly extended with an empty margin (half the size of the lookup-table) around the borders before running the reconstruction. An isotropic voxel size of 1 mm was chosen. The reconstructions are saved in a 16-bit NIfTI format and visualized and investigated using 3D Slicer (The Slicer Community). The look-up table used for the MLEM reconstruction was created robotically in a wet lab setting. To this end, a point source was created, using 100 µL FerroTrace (5.5 mg Fe), placed at the tip of a 200 µL Eppendorf tube (Eppendorf, Hamburg, Germany). Assuming rotational symmetry, half a slice through the middle of the look-up table (plane x-z, with z pointing down the longitudinal axis of the detector) was acquired. This was done by moving the robot arm to the starting position and start the acquisition of the point source measurements, taking about 6 measurements before moving to the next spot on a 25 × 40 mm x-z grid, with a step size of 1 mm. The resulting data was rotated around the z axis to create a full 3D look-up table. Afterwards, the look-up table was smoothed with a 3 mm Gaussian filter and rescaled to 60% to reduce artificial broadening introduced by the filter.

### Surface imaging and hybrid MPI/SI

To provide anatomical reference for the MPI scan with respect to the phantom surface (e.g., the skin), a 3D model of the phantom was created using optical surface imaging, similar as investigated for SPECT imaging of surgical specimens^53,54^. To this end, a handheld Artec Eva^®^ LiDAR scanner was used (Artec3D^®^, Luxembourg; 0.5 mm resolution; dimensions: 262 × 158 × 64 mm) for acquisition, while 3D model reconstruction was performed in Artec Studio 17. The surface model was exported as Wavefront OBJ file and visualized and manually fused with the MPI scan in 3D Slicer software.

### Proof-of-concept and system characterization phantoms

In the proof-of-concept vascular phantom, both arteries consisted of silicon tubing with a length of 90 mm and an inner diameter of 3.2 mm, inserted in an empty plastic mannequin human-sized head. They were filled with a concentration of 27.5 mg/mL, providing the non-obstructed vessel with roughly 19 mg, and the obstructed vessel with roughly 14 mg.

System sensitivity and depth detection were measured three times (taking the average) in a single combined setup. Here, Eppendorf tubes were filled with decreasing amount of FerroTrace, starting with 55 mg of Fe content, diluted down with saline to 3.4 µg of Fe content. Subsequently, for every amount of contrast agent, the system depth of detection was determined by moving the tube away from the detector surface until no longer observable; effectively mapping both the system detection depth for various amounts of contrast agent, as well as the system sensitivity at each depth.

Resolvability was investigated at shallow depth (within roughly 5 mm) by varying the distance (edge-to-edge) between 2 glass capillaries (0.8 mm inside diameter; 1.7 mm outside diameter) filled with 17 µL of FerroTrace (i.e., 0.9 mg). The distance between the capillaries was varied from 30 mm down to 5 mm, making an MPI reconstruction for every capillary-to-capillary distance; effectively mapping the smallest distance at which the individual capillaries were still distinguishable within the acquired MPI scan.

## Data Availability

The datasets generated and/or analyzed during the current study are available from the corresponding author, M.N. van Oosterom, m.n.van_oosterom@lumc.nl, upon reasonable request.

## Code availability

The MLEM reconstruction pipeline described in this study is publicly available via Zenodo (DOI: 10.5281/zenodo.21108560). The repository contains the reconstruction pipeline together with an example dataset.
